# Predictors of skin self-examination before and after a melanoma diagnosis: the role of medical advice and patient’s level of education

**DOI:** 10.1186/1755-7682-6-8

**Published:** 2013-02-27

**Authors:** Annett Körner, Adina Coroiu, Claudia Martins, Beatrice Wang

**Affiliations:** 1 Department of Educational and Counselling Psychology, McGill University, Montréal, Canada; 2 Lady Davis Institute for Medical Research-Jewish General Hospital, Montréal, Canada; 3 Louise-Granofsky-Psychosocial Oncology Program, Segal Cancer Centre, Montréal, Canada; 4 Psychosocial Oncology Program, McGill University Health Centre, Montréal, Canada; 5 Department of Gynecology-Oncology, McGill University Health Centre, Montréal, Canada; 6 Department of Dermatology, McGill University, Montréal, Canada; 7 Melanoma Clinic, McGill University Health Centre, Montréal, Canada

**Keywords:** Melanoma, Skin self-exam, Medical advice, Education level

## Abstract

**Background:**

Cutaneous melanoma is the fastest growing tumor of the skin and the median life span of patients with advanced disease is less than a year. Melanoma-related mortality can be reduced through early detection via clinical skin exams and patient self-examination. Despite the potential to reducing the medical burden associated with clinical skin exams, systematic and regular skin self-examinations (SSE) are rarely performed. The current study examined psychosocial predictors of SSE and changes in SSE behavior from pre- to post-diagnosis in order to guide future melanoma prevention initiatives.

**Findings:**

A consecutive sample of 47 melanoma survivors was drawn from a tertiary care clinic. Most melanomas had been detected by patients, spouses and other laypersons. Higher education was related to more frequent SSE at pre-diagnosis, more thorough SSE at post-diagnosis, and more frequent reports of having been advised to perform SSE at post-diagnosis. SSE behaviors increased significantly from pre- to post-diagnosis.

**Conclusions:**

These findings suggest that different patient subgroups display varied knowledge base, readiness for change, and receptiveness for medical advice. Thus, interventions seeking to enhance skin self-exam practice may be most effective when the individual’s psychosocial characteristics are taken into account.

## Background

Cutaneous melanoma is the deadliest and fastest growing tumor of the skin; it develops with a significant detectable preclinical phase, making it conducive to detection while in its curable stage; finally, the majority of melanomas are self-detected by patients and skin self-examination (SSE) has been shown to result in earlier diagnosis and significantly lower melanoma-related mortality [1]. The American Academy of Dermatology, the Canadian Dermatology Association, and the American Cancer Society recommend monthly, whole body SSE for high-risk populations, such as melanoma survivors. Despite this, the prevalence of systematic and regular SSE is low [2-4]. Further, clinical care guidelines [5-7] recommend the practice of monthly SSE among melanoma survivors; however, up to 70% of melanoma patients indicate that they have never been advised to self-examine their body [3].

Previous research on demographic predictors of SSE yielded mixed results (see [8,9] for comprehensive reviews), e.g., linking both younger and older age and both lower and higher educational statuses to better practice of SSE. Further, despite the fact that men are a critical subgroup presenting with more advanced melanoma and higher mortality rates, previous findings on the effect of sex upon SSE were inconclusive, reporting either no sex differences or more SSE in women [8,9]. Finally, being informed about SSE by one’s physician or nurse was associated with more frequent SSEs [10].

The current pilot study examined the predictors of SSE, for which previous research yielded inconclusive results, in order to further inform the first, large-scale melanoma prevention study in Canada, currently in progress at the Health Psychology Research Group at McGill University. The present study had two objectives: (1) to examine socio-demographic (i.e., age, gender, education) and medical predictors (i.e., melanoma stage) of SSE practice at pre- and post-diagnosis; and (2) to assess the changes in SSE practice from pre- to post-diagnosis among melanoma patients.

## Method

The study protocol was approved by the Research Ethics Office of the McGill University Health Centre (reference no. 10-126-PSY). Fourty-seven consecutive patients with cutaneous melanoma consented to participate in this study at a clinic affiliated with McGill University. Socio-demographic variables were assessed via a brief self-report questionnaire. SSE-related behaviour was assessed with four questions (see Table 1) inquiring about the practice of SSE and about having been advised to perform SSE a) pre- and b) post-diagnosis of melanoma. The melanoma stage was retrieved from the medical charts. Logistic regressions were conducted with the SSE items as the dependent variable and socio-demographic and medical variables as covariates. McNemar analyses were conducted to test the percentage increase in the SSE-related behaviors from pre-diagnosis to post-diagnosis.

**Table 1:** SSE practice at pre- and post-diagnosis

**SSE Items**	**% *(n)***	
	**PRE**	**POST**
1. Did you ever self-examine your skin for suspicious changes?		
**YES**	**39.02 (16)**	**87.80 (36)**
NO	61.00 (25)	12.20 (5)
2. Did you ever use a melanoma picture to help with the skin exam?		
**YES**	**9.76 (4)**	**24.39 (10)**
NO	90.24 (37)	75.61 (31)
3. Did you ever have someone else help you examine your skin?		
**YES**	**28.57 (12)**	**57.14 (24)**
NO	71.41 (30)	42.86 (18)
4. Have you ever been advised by a doctor or nurse to self-examine your skin?		
**YES**	**36.59 (15)**	**70.73 (29)**
NO	63.41 (26)	29.27 (12)

## Results

The descriptive statistics for the socio-demographic and medical variables are shown in Table 2, e.g., documenting that most melanomas were first detected by patients themselves, partners/spouses and other laypersons. Higher education was associated with an increased likelihood of a) having ever self-examined the skin for suspicious lesions at pre-diagnosis (*OR* = 1.29; 95% CI [1.04; 1.60]; *p* = .021; Negelkerke *R*^*2*^ = 18%); b) having used a melanoma picture to assist with SSE post-diagnosis (*OR* = 1.38; 95% CI [1.03; 1.85]; *p* = .031; Negelkerke *R*^*2*^ = 19%); and c) having been advised to perform SSE by a health care professional at post-diagnosis (*OR* = 1.27; 95% CI [1.00; 1.61]; *p* = .054; Negelkerke *R*^*2*^ = 14%). Age and gender were not significantly related to any of the four SSE-related behaviours at pre- or post-diagnosis. A more advanced melanoma stage was associated with an increased likelihood of a) having used a melanoma picture during SSE at post-diagnosis (*OR* = 2.34; 95% CI [1.20; 4.57]; *p* = .013; Negelkerke *R*^*2*^ = 26%); and b) having had someone else assist with SSE at post-diagnosis (*OR* = 2.38; 95% CI [1.18; 4.81]; *p* = .015; Negelkerke *R*^*2*^ = 25%). Across the entire sample, the response rate for the SSE behavior was higher at pre-diagnosis (i.e., ranging from 46 to 47 participants) than at post-diagnosis (ranging from 41 to 43 participants). A 4 × 2 table including paired SSE-related behaviors at pre-and post-diagnosis is presented in Table 2. Sample sizes varied due to listwise deletion, which was required given that McNemar analysis is a paired-sample test. From pre- to post-diagnosis, there was a significant increase in a) the general practice of SSE (χ^2^ = 18.18, *p* < .001), b) having been assisted by someone else in SSE (χ^2^ = 12, *p* = .0005), and c) having been advised on SSE by a health care professional (χ^2^ = 9.80, *p* = .0017). The increase from pre- to post-diagnosis in using a melanoma picture during self-exams was marginally significant (χ^2^ = 3.60, *p* = .0578).

**Table 2:** Sample description (N = 47)

**Variable**	**M (*SD *)**	**% (*n *)**
Age in years	55.39 (12.62)	
Education in years since grade 1	13.03 (3.48)	
Sex		
Male		51.1 (24)
Female		48.9 (23)
Marital status		
Married		61.7 (29)
Common law		10.6 (5)
Single, never married		6.4 (3)
Divorced/separated		12.8 (6)
Widowed		8.5 (4)
Time since diagnosis in months		
<1		17.0 (8)
1 to 2		14.9 (7)
3 to 6		34.0 (16)
> 6		31.9 (15)
Missing		2.1 (1)
Melanoma stage		
In situ (stage 0)		40.4 (19)
Stage I		10.6 (5)
Stage II		14.9 (7)
Stage III		17.0 (8)
Missing		17.0 (8)
Melanoma detection		
Patient		42.6 (20)
Physician		10.6 (5)
Partner/spouse		12.8 (6)
Other (e.g., sister, offspring)		8.5 (4)
Missing		25.5 (12)

## Discussion

Education as one of the main social determinants of health (http://www.euro.who.int/__data/assets/pdf_file/0005/98438/e81384.pdf) was found to be a key predictor of SSE in this study. Individuals with higher education more often self-examined their skin for suspicious lesions before being diagnosed with melanoma, which confirms previous melanoma research findings [11] and further builds on a growing body of literature linking higher education with healthier life choices and less risky behaviours [12]. As education and socioeconomic status are interrelated, this result further corroborates previous research demonstrating that individuals with lower socioeconomic status are burdened with higher melanoma-related mortality [13]. The fact that this study did not find an association at pre-diagnosis between educational level and the use of a melanoma picture or the use of someone’s help during SSE suggests that even higher educated individuals may be lacking the more specific knowledge about how to conduct effective SSE. At post-diagnosis, higher education was related to more thorough SSE, i.e., by use of a melanoma picture, and increased self-reports of having been advised to perform SSE. Although recall bias may partially explain these results, they may also indicate a better learning aptitude amongst higher educated individuals, who tend to seek medical advice more actively. Consequently, SSE instructions may be more readily provided to such patients. Nevertheless, in the current sample, all SSE behaviors improved from pre- to post-diagnosis with 71% of the patients reporting at post-diagnosis having received advice regarding SSE and 88% having performed at least one self-exam. The generalizability of these findings should be interpreted with caution due to the relatively small sample size; the cross-sectional design, and the fairly global assessment of SSE practice; also, the high endorsement rates of SSE behavior may have masked gender and age differences. However, these findings speak to 1) clinicians’ adherence to care guidelines, and 2) patients’ willingness for behavioral change. Further, the current study suggests that future intervention programs seeking to enhance SSE practice may be more effective if socio-demographic characteristics (e.g., educational status) of the target population are taken into account [14,15].

## Consent

Written informed consent to use these data for publication purposes was obtained from all study participants.

## Abbreviations

SSE: Skin self-examination.

## Competing interests

The authors declare that they have no competing interests.

## Authors’ contributions

AK designed and implemented this study overseeing data acquisition and analysis. AC analyzed the data. AK and AC interpreted the findings and wrote the manuscript. BW and CM contributed to data collection and manuscript revisions. All authors read and approved the final manuscript.
